# Perampanel Affects Up-Stream Regulatory Signaling Pathways of GluA1 Phosphorylation in Normal and Epileptic Rats

**DOI:** 10.3389/fncel.2019.00080

**Published:** 2019-03-01

**Authors:** Ji-Eun Kim, Hui-Chul Choi, Hong-Ki Song, Tae-Cheon Kang

**Affiliations:** ^1^Department of Anatomy and Neurobiology, College of Medicine, Hallym University, Chuncheon, South Korea; ^2^College of Medicine, Institute of Epilepsy Research, Hallym University, Chuncheon, South Korea; ^3^Department of Neurology, College of Medicine, Hallym University, Chuncheon, South Korea

**Keywords:** BIM, CAMKII, cyclosporin A, ERK1/2, H-89, JNK, KN-93, okadaic acid

## Abstract

To elucidate the pharmacological properties of perampanel [2-(2-oxo-1-phenyl-5-pyridin-2-yl-1,2-dihydropyridin-3-yl)benzonitrile, a novel non-competitive antagonist of AMPA receptor], we investigated its effects on the up-stream regulatory pathways of GluA1 phosphorylation including protein kinase C (PKC), Ca^2+^-calmodulin-dependent protein kinase II (CAMKII), protein kinase A (PKA), extracellular signal-regulated kinase 1/2 (ERK1/2), c-Jun N-terminal kinase (JNK), protein phosphatase (PP) 1, PP2A, and PP2B in normal and pilocarpine-induced epileptic rat model using Western blot analysis. In normal animals, perampanel affected GluA1 expression/phosphorylation, PKC, CAMKII, PKA, ERK1/2, JNK, and PPs activities. In epileptic rats, perampanel effectively inhibited spontaneous seizure activities. Perampanel enhanced phospho (p)-GluA1-S831 and -S845 ratios (phosphoprotein/total protein), while it reduced GluA1 expression. Perampanel also increased pCAMKII and pPKA ratios, which phosphorylate GluA1-S831 and -S845 site, respectively. Perampanel elevated pJNK and pPP2B ratios, which phosphorylates and dephosphorylates both GluA1-S831 and -S845 sits. Perampanel also increased pERK1/2 ratio in epileptic animals, while U0126 (an ERK1/2 inhibitor) did not affect pGluA1 ratios. Perampanel did not influence PKC, PP1, and PP2A expression levels and their phosphorylation ratios. In addition, perampanel did not have a detrimental impact on cognitive abilities of epileptic and normal rats in Morris water maze test. These findings suggest that perampanel may regulate AMPA receptor functionality via not only blockade of AMPA receptor but also the regulations of multiple molecules (CAMKII, PKA, JNK, and pPP2B)-mediated GluA1 phosphorylations without negative effects on cognition, although the effects of perampanel on PKC, PP1, and PP2A activities were different between normal and epileptic rats.

## Introduction

Glutamate is one of the excitatory neurotransmitters, which is involved in various physiological functions of the brain, including synaptic plasticity. The glutamate action is mediated by ionotropic and metabotropic receptors (Greenamyre and Porter, 1994; Michaelis, 1998). α-Amino-3-hydroxy-5-methyl-4-isoxazolepropionic acid (AMPA) receptor is one of ligand-gated ion channels for glutamate (Traynelis et al., 2010). AMPA receptors have four subunits (GluA1–GluA4), which compose the pentameric structures of the receptor (Borges and Dingledine, 1998; Dingledine et al., 1999). AMPA receptors influx Na^+^ ion and, to a lesser extent, Ca^2+^ (Hollmann et al., 1991). AMPA receptor plays a role in fast postsynaptic depolarization, and is critical to seizure initiation, epileptic synchronization and the spread of seizure activity. Thus, pharmacological inhibitors of AMPA receptors may have potentials as a therapeutic approach for the treatment of epilepsy (Rogawski, 2013).

Perampanel [2-(2-oxo-1-phenyl-5-pyridin-2-yl-1,2-dihydropyridin-3-yl)benzonitrile] is a novel non-competitive antagonist of AMPA receptor that has been approved by the European Medicines Agency and the U.S. Food and Drug Administration as an add-on treatment for partial-onset seizures in patients >12 years. Furthermore, perampanel demonstrates broad-spectrum anti-seizure effects in preclinical animal models, without affecting the *N*-methyl-*D*-aspartate (NMDA) or kainate receptors (Hanada et al., 2011; Ceolin et al., 2012; Krauss, 2013; Steinhoff et al., 2013; Chen et al., 2014). Although perampanel inhibits AMPA receptor-mediated currents, its effects on signaling pathways concerning AMPA activity remain to be elucidated.

The phosphorylation of GluA1 subunits increases the conductance of AMPA receptor and potentiates rapid excitatory neurotransmission (Derkach et al., 1999). The carboxyl terminus of GluA1 subunit at serine (S) residues 831 (S831) and 845 (S845) are phosphorylated by protein kinases (Snyder et al., 2000). S831 site is phosphorylated by protein kinase C (PKC), Ca^2+^-calmodulin-dependent protein kinase II (CAMKII) and c-Jun N-terminal kinase (JNK). In contrast, phosphorylation of S845 is regulated by protein kinase A (PKA), extracellular signal-regulated kinase 1/2 (ERK1/2), and JNK (Banke et al., 2000; Wang et al., 2006; Ahn and Choe, 2009). In addition, various protein phosphatases (PPs) also regulate AMPA receptor functionality by dephosphorylating these sites (Snyder et al., 2003; Ahn and Choe, 2009). With respect to these previous reports, it is likely that perampanel may modulate GluA1 phosphorylation via regulating activities of protein kinases and phosphatases. Therefore, the objective in the present study is to investigate the intracellular mechanisms of perampanel involving GluA1 phosphorylation in normal and epileptic rats.

## Materials and Methods

### Experimental Animals, Chemicals and Experimental Design

**FIGURE 1:**
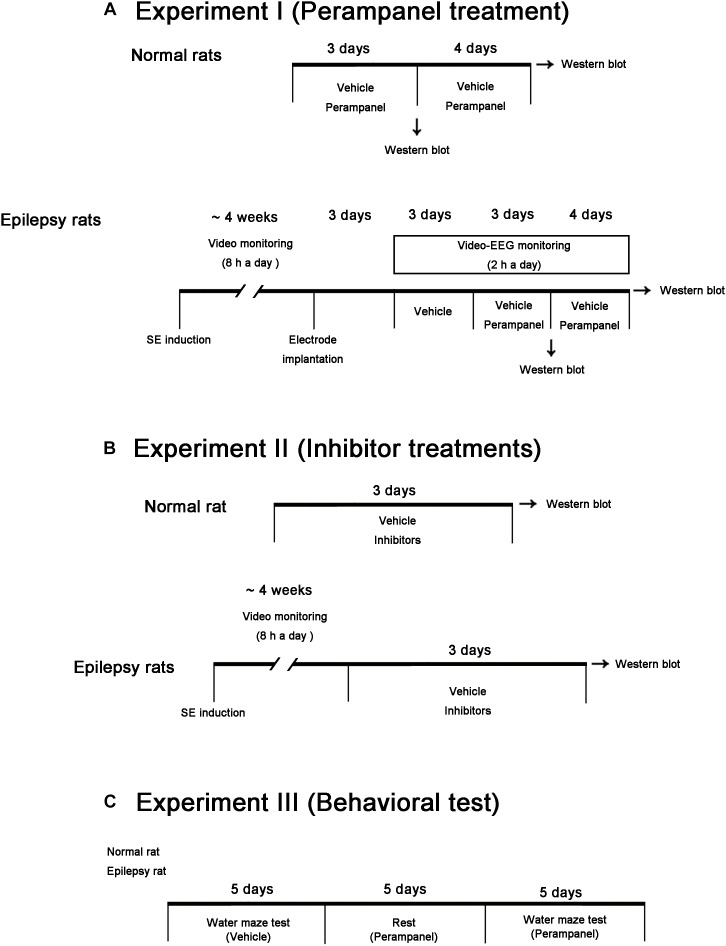
Scheme of the experimental designs in the present study. **(A)** Studies for evaluation of the effects of perampanel on expressions/phosphorylations of GluA1, kinases and PPs in normal and epileptic rats. **(B)** Studies for validation of the effects of kinase and PP inhibitors on GluA1 expression and phosphorylations in normal and epileptic rats. **(C)** Protocols for measurement of cognitive effects of perampanel by water maze test.

In the present study, we used male Sprague-Dawley (SD) rats (7 weeks old) obtained from the Daehan Biolink, South Korea. Animals were given a commercial diet and water *ad libitum* under controlled conditions (22 ± 2°C, 55 ± 5% and a 12:12 light/dark cycle with lights). Animal protocols were approved by the Institutional Animal Care and Use Committee of Hallym University (Chuncheon, South Korea). The number of animals used and their suffering were minimized in all cases. All reagents were obtained from Sigma-Aldrich (St. Louis, MO, United States), except as noted. Figure 1 illustrates the scheme of the experimental design of methodology and the animal numbers used in the present study (Figure 1).

### SE Induction

Rats were given LiCl (127 mg/kg, i.p.) 24 h before the pilocarpine treatment. Animals were treated with pilocarpine (30 mg/kg, i.p.) 20 min after atropine methylbromide (5 mg/kg i.p.). Two hours after SE onset, diazepam (Valium; Hoffmann-la Roche, Neuilly-sur-Seine, France; 10 mg/kg, i.p.) was administered to terminate SE and repeated, as needed. Control animals received saline in place of pilocarpine. Animals were video-monitored 8 h a day for selecting chronic epileptic rats showing spontaneous recurrent seizures (Ko and Kang, 2015). Behavioral seizure severity was evaluated according to Racine’s scale: 1, immobility, eye closure, twitching of vibrissae, sniffing, facial clonus; 2, head nodding associated with more severe facial clonus; 3, clonus of one forelimb; 4, rearing, often accompanied by bilateral forelimb clonus; and 5, rearing with loss of balance and falling accompanied by generalized clonic seizures. We classified epileptic rats that showed behavioral seizure activities with seizure score ≥3 more than once.

### Electrode Implantation

Control and epileptic rats were implanted with monopolar stainless steel electrodes (Plastics One, Roanoke, VA, United States) in the right hippocampus under Isoflurane anesthesia (3% induction, 1.5–2% for surgery, and 1.5% maintenance in a 65:35 mixture of N_2_O:O_2_) using the following coordinates: -3.8 mm posterior; 2.0 mm lateral; -2.6 mm depth. Throughout surgery, core temperature of each rat was maintained 37–38°C. Electrode was secured to the exposed skull with dental acrylic (Ko and Kang, 2015).

### Perampanel Trials and Quantification of Seizure Activity

After baseline seizure activity was determined over 3 days, perampanel (8 mg/kg, i.p, Eisai Korea Inc.) or saline (vehicle) was daily administered at a certain time of the day (PM 6:00) over a 3 days or a 1 week period. EEG was recorded 2 h a day at the same time (Figure 1). EEG signals were recorded with a DAM 80 differential amplifier (0.1–3000 Hz bandpass; World Precision Instruments, Sarasota, FL, United States) and the data were digitized (1000 Hz) and analyzed using LabChart Pro v7 (ADInstruments, NSW, Australia). Behavioral seizure severity was also evaluated as aforementioned. After recording (18 h after the last treatment), animals were used for western blot study. Some animals (*n* = 4) were anesthetized with urethane (1.5 g/kg, i.p.) and then transcardially perfused with 4% paraformaldehyde (pH 7.4). The brains were removed and post-fixed overnight in the same solution then sequentially placed in 30% sucrose at 4°C. Coronal sections were cut at a thickness of 30 μm on a cryostat, and used for Cresyl violet staining to further confirm epileptic animals.

### Chemical Infusion

Under Isoflurane anesthesia (3% induction, 1.5–2% for surgery and 1.5% maintenance in a 65:35 mixture of N_2_O:O_2_), animals were infused each chemical into the right lateral ventricle (1 mm posterior; 1.5 mm lateral; -3.5 mm depth to the bregma) with a brain infusion kit 1 and an ALZET 1003D osmotic pump (ALZET, Cupertino, CA, United States). Osmotic pump contained (1) vehicle, (2) bisindolylmaleimide (BIM; a PKC inhibitor, 25 μM), (3) KN-93 (a CAMKII inhibitor, 25 μM, Santa Cruz, United States), (4) H-89 (a PKA inhibitor, 10 μM), (5) U0126 (an ERK1/2 inhibitor, 25 μM), (6) SP600125 (a JNK inhibitor, 10 μM), (7) okadaic acid (a PP1/PP2A inhibitor, 10 μM, Cayman, United States), or (8) cyclosporin A (CsA, a PP2B inhibitor, 250 μM). In pilot study and our previous studies, each compound treatment did not show behavioral and neurological defects and could not change the seizure susceptibility and seizure severity in response to pilocarpine in normal animals (Min et al., 2017; Park and Kang, 2018). Three days after infusion, animals were used for western blot.

### Western Blot

After animals were sacrificed via decapitation, the left hippocampus was obtained. The hippocampal tissues were homogenized, and determined protein concentration using a Micro BCA Protein Assay Kit (Pierce Chemical, Rockford, IL, United States). Western blot was performed by the standard protocol. The primary antibodies used in the present study were listed in Supplementary Table 1. The bands were detected and quantified on ImageQuant LAS4000 system (GE Healthcare, Piscataway, NJ, United States). Since ERK1/2 and pERK1/2, but not others, antibodies clearly showed two (p42 and p44) bands and were changed to the same degree, we quantified both bands. As an internal reference, rabbit anti-β-actin primary antibody (1:5000) was used. The values of each sample were normalized with the corresponding amount of β-actin. The ratio of phosphoprotein to total protein was described as phosphorylation ratio.

### Morris Water Maze

Spatial learning and memory were tested by the Morris water maze hidden platform task using the same maze and protocol as previous reported (Postina et al., 2004; Kim et al., 2016). Briefly, rats received five consecutive days of hidden platform training. The animals were allowed to search for the hidden platform for a period of 120 s. In the last day, animals were tested in a probe trial in which the platform was removed from the pool and allowed to search for a period of 120 s. Swimming time and path length in target quadrant, where the platform had been placed, were recorded.

### Data Analysis

One-way ANOVA with *post hoc* Bonferroni’s multiple comparison test (Biochemical data), Kruskal–Wallis test with Dunn’s multiple comparison test (seizure frequency and seizure severity), two-way ANOVA with *post hoc* Bonferroni’s multiple comparison test (probe trials of Morris water maze test), repeated measures two-way ANOVA with the least significant difference test (Morris water maze test), *χ*^2^ test (comparison of seizure frequency between responder and non-responder) and Student’s *t*-test (comparison of seizure duration between responder and non-responder) were used to determine statistical significance of data. A *p*-value below 0.05 was considered statistically significant.

## Results

### Effect of Perampanel on Spontaneous Seizure Activity in Epileptic Animals

**FIGURE 2:**
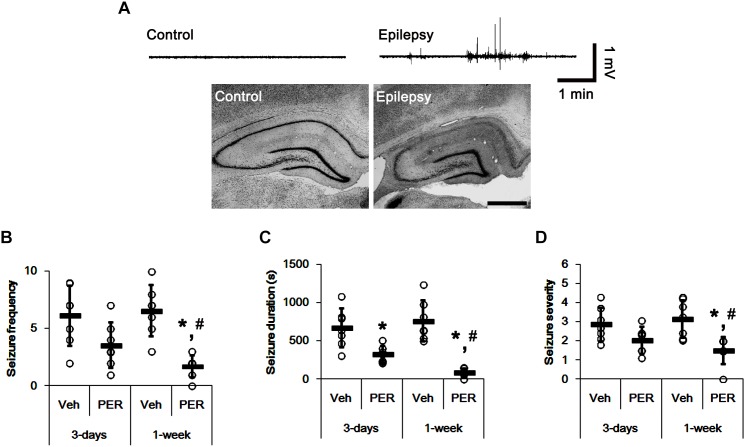
The effect of perampanel on spontaneous seizure activity in epileptic rats. **(A)** Representative EEG traces and hippocampal images obtained from normal and epileptic rats. Bar = 400 μm. **(B–D)** Quantitative values of seizure frequency **(B)**, total seizure duration **(C)**, and seizure severity **(D)** during 2 h of recording a day. Open circles indicate each individual value. Horizontal bars indicate mean value. Error bars indicate SD (*^∗,^*^#^*p* < 0.05 vs. vehicle and 3-days perampanel treatment, respectively; *n* = 7, respectively).

In the present study, normal (control) animals did not show behavioral or EEG seizure activity (Figure 2A). Perampanel did not affect behavior and EEG activity in normal animals (data not shown). During the recording of baseline seizure activities, epileptic rats showed that the seizure frequency was 6.14 ± 2.61/recording session (2 h) and the total seizure duration was 671.14 ± 253.79 s. The seizure severity (behavioral seizure core) was 2.87 ± 0.89 (Figure 2B–D). There was no difference in baseline seizure activities between vehicle- and perampanel-treated groups of epileptic rats (data not shown). In 3-days perampanel-treated group of epileptic rats (*n* = 7), animals showed a reduction of seizure duration [*F*_(1,12)_ = 10.8; *p* < 0.05, respectively; *n* = 7, respectively; Figure 2B–D], but not its frequency and severity [*F*_(1,12)_ = 4.3 and 3.75, respectively; Figure 2B–D]. In 1-week perampanel-treated group of epileptic rats (*n* = 10), rats showed the absence of seizures (*n* = 1) or the significant reduction in seizure activity (*n* = 6). The seizure frequency was 1.71 ± 0.95/recording session [*F*_(1,12)_ = 28.2; *p* < 0.05 vs. vehicle, *n* = 7, respectively; Figure 2B] and the total seizure duration was 91 ± 53.91 s [*F*_(1,12)_ = 42.55; *p* < 0.05 vs. vehicle *n* = 7, respectively; Figure 2C]. The seizure severity was 1.5 ± 0.71 [*F*_(1,12)_ = 13.24; *p* < 0.05 vs. vehicle, *n* = 7, respectively; Figure 2D]. Three of ten rats showed that seizure frequency was unaltered by perampanel treatment (non-responders, *χ*^2^ = 0.08, 0.22, and 0.6, respectively; *p* = 0.78, 0.63, and 0.43, respectively). The seizure durations in non-responder group were also unchanged by perampanel. Non-responders were selected and omitted from the data analysis and the biochemical study.

### Effects of Perampanel on GluA1 Expression and Its Phosphorylations

**FIGURE 3:**
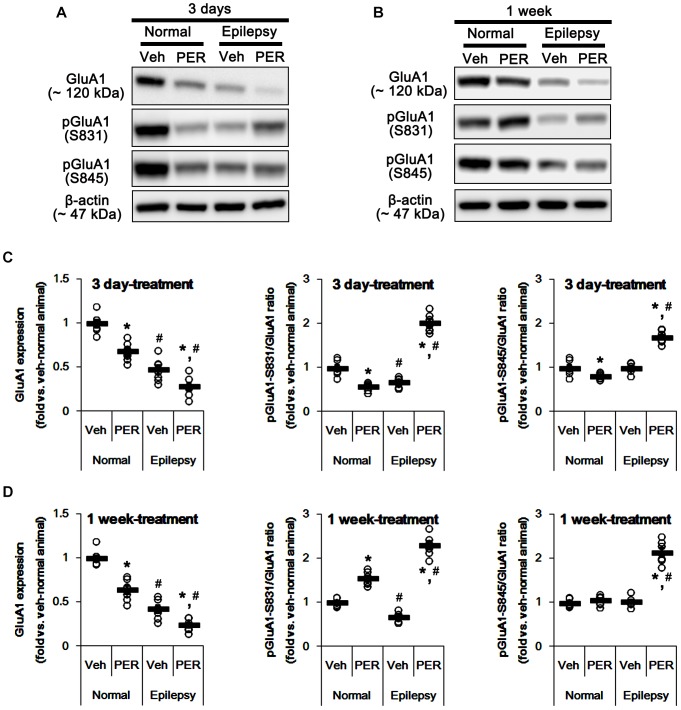
Effects of perampanel on GluA1 expression and its phosphorylations. **(A,B)** Representative images for western blot of GluA1, phospho (p)-GluA1-S831, and pGluA1-S845 in the hippocampal tissues obtained from 3-days **(A)** and 1-week treated groups **(B)**. **(C,D)** Quantifications of GluA1, pGluA1-S831, and pGluA1-S845 levels in 3-days **(C)** and 1-week treated groups **(D)**. Open circles indicate each individual value. Horizontal bars indicate mean value. Error bars indicate SEM (*^∗,^*^#^*p* < 0.05 vs. vehicle-treated animals and normal animals, respectively; *n* = 7, respectively).

Next, we investigated whether perampanel affects GluA1 expression and its phosphorylations. In normal animals, perampanel reduced GluA1 expressions to 68 and 64% of vehicle level in 3-days and 1-week treated group, respectively [*F*_(1,12)_ = 31.8 and 40.48, respectively; *p* < 0.05 vs. vehicle, *n* = 7, respectively; Figure 3A–D and Supplementary Figure 1]. pGluA1-S831 ratio was diminished to 58% of vehicle level in 3-days treated group [*F*_(1,12)_ = 35.49; *p* < 0.05 vs. vehicle, *n* = 7, respectively; Figure 3A–D], while it was enhanced to 1.5-fold of vehicle level in 1-week treated group [*F*_(1,12)_ = 70.97; *p* < 0.05 vs. vehicle, *n* = 7, respectively; Figure 3A–D]. pGluA1-S845 ratio was decreased to 80% of vehicle level in 3-days treated group [*F*_(1,12)_ = 8.09; *p* < 0.05 vs. vehicle, *n* = 7, respectively; Figure 3A–D], while it was recovered to vehicle level in 1-week treated group [*F*_(1,12)_ = 1.46, *n* = 7, respectively; Figure 3A–D].

As compared to normal rats, epileptic rats showed the reduction in GluA1 expression [∼45% of vehicle-treated normal rat level, *F*_(1,__26)_ = 184.92; *p* < 0.05, *n* = 14, respectively; Figure 3A–D] and pGluA1-S831 ratio [∼67% of vehicle-treated normal rat level, *F*_(1,__26)_ = 57.41; *p* < 0.05, *n* = 14, respectively; Figure 3A–D] without changing pGluA1-S845 ratio [*F*_(1,__26)_ = 0.0002, *n* = 14, respectively; Figure 3A–D]. Perampanel reduced GluA1 expressions to 28 and 24% of vehicle-treated normal rat level in 3-days and 1-week treated group, respectively [*F*_(1,12)_ = 8.28 and 14.54, respectively; *p* < 0.05 vs. vehicle, *n* = 7, respectively; Figure 3A–D]. However, perampanel increased pGluA1-S831 ratios to 2.02- and 2.31-fold of vehicle-treated normal rat level in 3-days and 1-week treated group, respectively [*F*_(1,12)_ = 243.83 and 297.86, respectively; *p* < 0.05 vs. vehicle, *n* = 7, respectively; Figure 3A–D]. Similarly, pGluA1-S845 ratios were increased to 1.69- and 2.49-fold of vehicle-treated normal rat level by 3-days and 1-week over perampanel treatment, respectively [*F*_(1,12)_ = 106.34 and 120.59, respectively; *p* < 0.05 vs. vehicle, *n* = 7, respectively; Figure 3A–D]. Since GluA1 phosphorylations represent the enhanced AMPA receptor-mediated synaptic currents (Zhang et al., 2016), our findings indicate that alterations in pGluA1-S831 and -S845 ratios may be adaptive responses for the reductions in AMPA receptor functionality or GluA1 expression level by perampanel.

### Effects of Perampanel on PKC and CAMKII Phosphorylation

**FIGURE 4:**
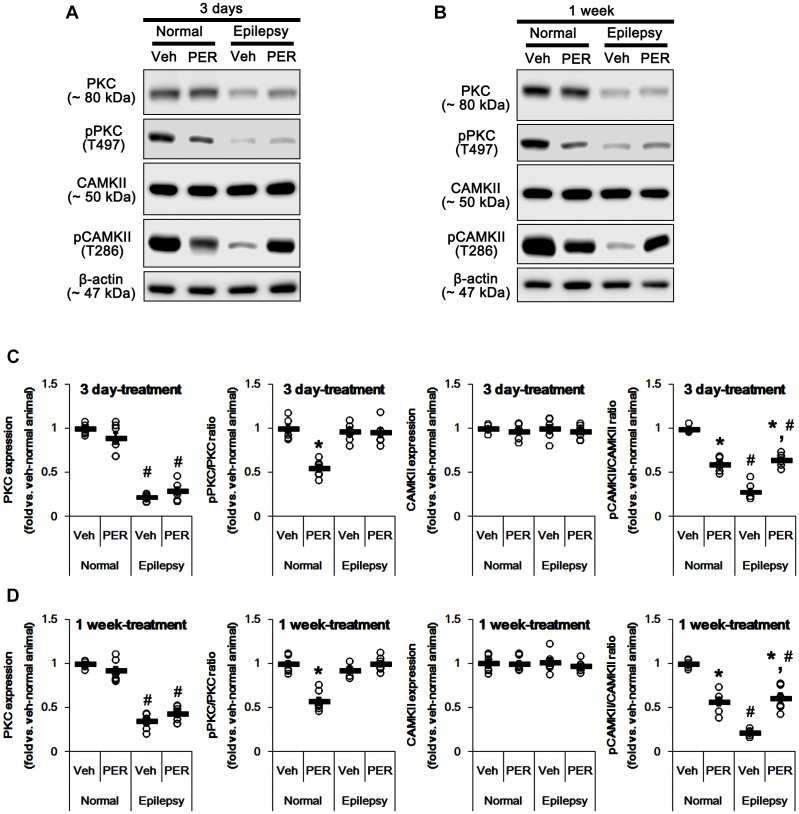
Effects of perampanel on PKC and CAMKII expressions and their phosphorylations. **(A,B)** Representative images for western blot of PKC, phospho (p)-PKC, CAMKII, and pCAMKII in the hippocampal tissues obtained from 3-days **(A)** and 1-week treated groups **(B)**. **(C,D)** Quantifications of PKC, pPKC, CAMKII, and pCAMKII levels in 3-days **(C)** and 1-week treated groups **(D)**. Open circles indicate each individual value. Horizontal bars indicate mean value. Error bars indicate SEM (*^∗,^*^#^*p* < 0.05 vs. vehicle-treated animals and normal animals, respectively; *n* = 7, respectively).

GluA-S831 site is present in a consensus phosphorylation site motif for PKC and CAMKII (Banke et al., 2000; Wang et al., 2006; Ahn and Choe, 2009). Thus, we explored the effects of perampanel on phosphorylations (activities) of PKC and CAMKII. In normal animals, perampanel reduced pPKC ratios to ∼ 55 and ∼ 57% of vehicle-treated normal rat level in 3-days and 1-week treated group, respectively [*F*_(1,12)_ = 69.66 and 61.14, respectively; *p* < 0.05 vs. vehicle, *n* = 7, respectively; Figure 4A– D and Supplementary Figure 2], without changing its expression level. Perampanel also decreased pCAMKII ratios to ∼ 60 and ∼ 57% of vehicle-treated normal rat level in 3-days and 1-week treated group, respectively, without altering its expression level [*F*_(1,12)_ = 117.98 and 93.89, respectively; *p* < 0.05 vs. vehicle, *n* = 7, respectively; Figure 4A–D].

In epileptic rats, PKC expression was decreased to ∼ 28% of vehicle-treated normal rat level [*F*_(1,26)_ = 690.54; *p* < 0.05, *n* = 14, respectively; Figure 4A–D]. However, pPKC ratio was similar to that in normal rats [*F*_(1,26)_ = 2.63; Figure 4A–D]. In contrast, pCAMKII ratio was decreased to ∼ 25% of vehicle-treated normal rat level [*F*_(1,26)_ = 1177.21; *p* < 0.05, *n* = 14, respectively; Figure 4A–D], while its expression was observed as normal level [*F*_(1,26)_ = 0.009; Figure 4A–D]. Unlike normal animals, perampanel did not influence PKC expression and pPKC ratio in 3-days [*F*_(1,12)_ = 3.48 and 0.02, respectively; *n* = 7, respectively; Figure 4A–D] and 1-week treated group of epileptic rats [*F*_(1,12)_ = 3.69 and 3.74, respectively; *n* = 7, respectively; Figure 4A–D]. However, perampanel enhanced pCAMKII ratios to ∼ 65 and ∼ 61% of vehicle-treated normal rat level in 3-days and 1-week treated group of epileptic rats, respectively [*F*_(1,12)_ = 72.96 and 60.03, respectively; *p* < 0.05 vs. vehicle, *n* = 7, respectively; Figure 4A–D], without affecting its expression [*F*_(1,12)_ = 0.41 and 0.84, respectively; *n* = 7, respectively; Figure 4A–D]. Considering the reduced PKC expression, it is likely that perampanel-mediated enhancement of pGluA1-S831 ratio in epileptic rats may be regulated by CAMKII rather than PKC.

**FIGURE 5:**
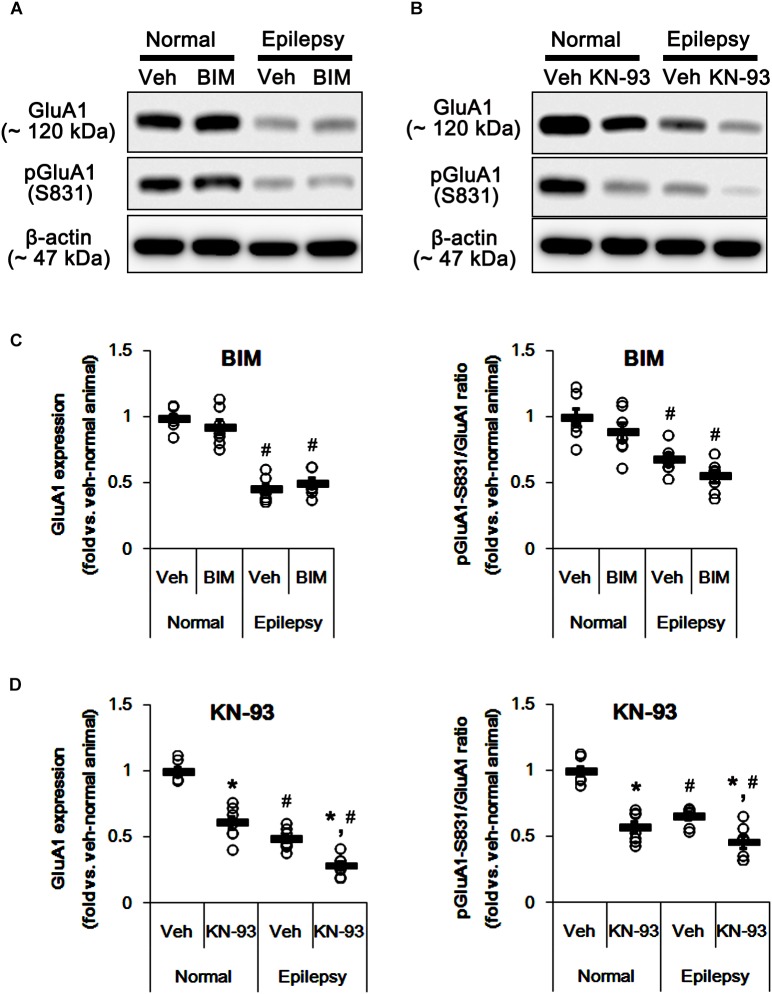
Effects of BIM and KN-93 on GluA expressions and their phosphorylations. **(A,B)** Representative images for western blot of GluA1, phospho (p)-GluA1-S831, and pGluA1-S845 in the hippocampal tissues obtained from 3-days infused groups **(B)**. **(C,D)** Quantifications of GluA1, pGluA1-S831, and pGluA1-S845 levels in BIM- **(C)** and KN-93 infused groups **(D)**. Open circles indicate each individual value. Horizontal bars indicate mean value. Error bars indicate SEM (*^∗,^*^#^*p* < 0.05 vs. vehicle-treated animals and normal animals, respectively; *n* = 7, respectively).

To confirm this, we evaluated the effects of BIM (a PKC inhibitor) and KN-93 (a CAMKII inhibitor) on GluA1 expression and its S831 phosphorylation in normal and epileptic rats. BIM did not affect GluA1 expression and pGluA1-S831 ratio in normal [*F*_(1,12)_ = 0.64 and 1.4, respectively; *n* = 7, respectively; Figure 5A,C and Supplementary Figure 3] and epileptic rats [*F*_(1,12)_ = 0.89 and 4.46, respectively; *n* = 7, respectively; Figure 5A,C]. However, KN-93 reduced GluA1 expression and pGluA1-S831 ratio in normal [*F*_(1,12)_ = 48.52 and 58.66, respectively; *n* = 7, respectively; Figure 5B,D and Supplementary Figure 3] and epileptic rats [*F*_(1,12)_ = 23.01 and 12.56, respectively; *n* = 7, respectively; Figure 5B,D]. Therefore, these findings also indicate that perampanel may regulate GluA1 expression and pGluA1-S831 ratio via CAMKII signaling pathway.

### Effects of Perampanel on PKA and ERK1/2 Phosphorylation

**FIGURE 6:**
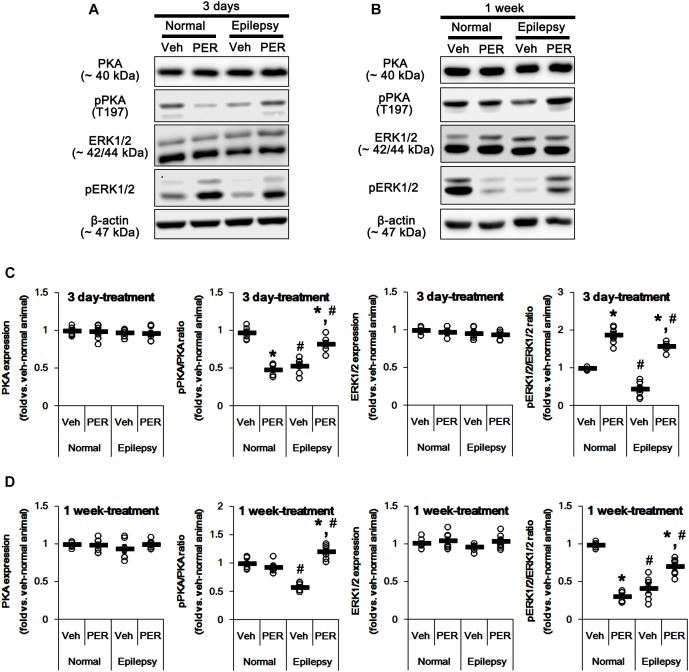
Effects of perampanel on PKA and ERK1/2 expressions and their phosphorylations. **(A,B)** Representative images for western blot of PKA, phospho (p)-PKA, ERK1/2, and pERK1/2 in the hippocampal tissues obtained from 3-days **(A)** and 1-week treated groups **(B)**. Both ERK1/2 and pERK1/2 antibodies clearly show two (p42 and p44) bands. **(C,D)** Quantifications of PKA, pPKA, ERK1/2, and pERK1/2 levels in 3-days **(C)** and 1-week treated groups **(D)**. Open circles indicate each individual value. Horizontal bars indicate mean value. Error bars indicate SEM (*^∗,^*^#^*p* < 0.05 vs. vehicle-treated animals and normal animals, respectively; *n* = 7, respectively).

Since both PKA and ERK1/2 phosphorylate GluA-S845 site (Banke et al., 2000; Wang et al., 2006; Ahn and Choe, 2009), we also tested whether perampanel changes phosphorylations (activities) of PKA and ERK1/2. In normal animals, PKA and ERK1/2 expressions were unaltered by 3-days [*F*_(1,12)_ = 0.06 and 1.03, respectively; *n* = 7, respectively; Figure 6A–D and Supplementary Figure 4] and 1-week over perampanel treatment [*F*_(1,12)_ = 0.1 and 0.46, respectively; *n* = 7, respectively; Figure 6A–D]. However, perampanel reduced pPKA ratio to 49% of vehicle-treated normal rat level in 3-days treated group [*F*_(1,12)_ = 166.3; *p* < 0.05 vs. vehicle, *n* = 7, respectively; Figure 6A–D], but not 1-week treated group [*F*_(1,12)_ = 1.46; *n* = 7, respectively; Figure 6A–D]. In addition, perampanel increased pERK1/2 ratio to 1.9-fold of vehicle level in 3-days treated group [*F*_(1,12)_ = 114.64; *p* < 0.05 vs. vehicle, *n* = 7, respectively; Figure 6A–D], but decreased it to 0.31-fold of vehicle level in 1-week treated group [*F*_(1,12)_ = 560.12; *p* < 0.05 vs. vehicle, *n* = 7, respectively; Figure 6A–D].

**FIGURE 7:**
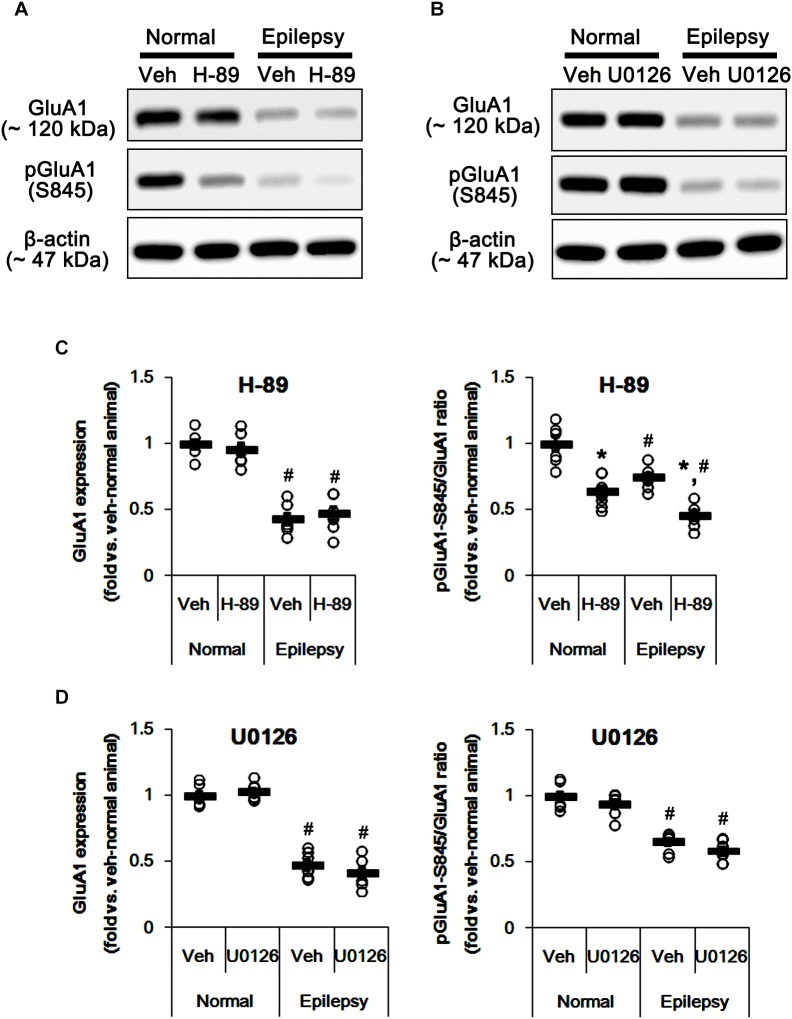
Effects of H-89 and U0126 on GluA expressions and their phosphorylations. **(A,B)** Representative images for western blot of GluA1, phospho (p)-GluA1-S831, and pGluA1-S845 in the hippocampal tissues obtained from 3-days infused groups **(B)**. **(C,D)** Quantifications of GluA1, pGluA1-S831, and pGluA1-S845 levels in H-89- **(C)** and U0126-infused groups **(D)**. Open circles indicate each individual value. Horizontal bars indicate mean value. Error bars indicate SEM (*^∗,^*^#^*p* < 0.05 vs. vehicle-treated animals and normal animals, respectively; *n* = 7, respectively).

In epileptic rats, pPKA and pERK1/2 ratios were decreased to ∼ 56 and ∼ 43% of vehicle-treated normal rat level without altering their expressions [*F*_(1,26)_ = 180.21 and 151.43, respectively; *p* < 0.05, *n* = 14, respectively; Figure 6A–D]. Perampanel did not affect PKA and ERK1/2 expressions in both 3-days [*F*_(1,12)_ = 0.006 and 0.17, respectively; *n* = 7, respectively; Figure 6A–D] and 1-week treated group of epileptic rats [*F*_(1,12)_ = 1.29 and 3.69, respectively; *n* = 7, respectively; Figure 6A–D]. However, perampanel elevated pPKA ratios were 83 and 121% of vehicle-treated normal rat level in 3-days [*F*_(1,12)_ = 30.4; *p* < 0.05 vs. vehicle, *n* = 7, respectively; Figure 6A–D] and 1-week treated group [*F*_(1,12)_ = 140.9; *p* < 0.05 vs. vehicle, *n* = 7, respectively; Figure 6A–D], respectively. Following perampanel treatment, pERK1/2 ratios were 158 and 71% of vehicle-treated normal rat level in 3-days [*F*_(1,12)_ = 175.99; *p* < 0.05 vs. vehicle, *n* = 7, respectively; Figure 6A–D] and 1-week treated group [*F*_(1,12)_ = 16.55; *p* < 0.05 vs. vehicle, *n* = 7, respectively; Figure 6A–D], respectively. These findings indicate that perampanel may differently affect PKA and ERK1/2 activities by treatment-length in normal animals. Regarding the effect of perampanel on GluA1-S845 ratio, our findings also suggest that PKA may phosphorylate GluA1-S845 rather than ERK1/2. Indeed, H-89 (a PKA inhibitor) reduced pGluA1-S845 ratios in normal [*F*_(1,12)_ = 27.18; *p* < 0.05 vs. vehicle, *n* = 7, respectively; Figure 7A,C and Supplementary Figure 5] and epileptic rats [*F*_(1,12)_ = 40.9; *p* < 0.05 vs. vehicle, *n* = 7, respectively; Figure 7A,C] without altering GluA1 expression levels [*F*_(1,12)_ = 0.51 and 0.36, respectively; *n* = 7, respectively; Figure 7A,C]. U0126 (an ERK1/2 inhibitor) did not affect GluA1 expressions and pGluA1-S845 ratios in normal [*F*_(1,12)_ = 0.81, and 1.61, respectively; *n* = 7, respectively; Figure 7B,D and Supplementary Figure 5] and epileptic rats [*F*_(1,12)_ = 1.43 and 2.89, respectively; *n* = 7, respectively; Figure 7B,D]. Thus, our findings indicate that perampanel may phosphorylate GluA1-S845 site by PKA activation in epileptic rats.

### Effects of Perampanel on JNK Phosphorylation

**FIGURE 8:**
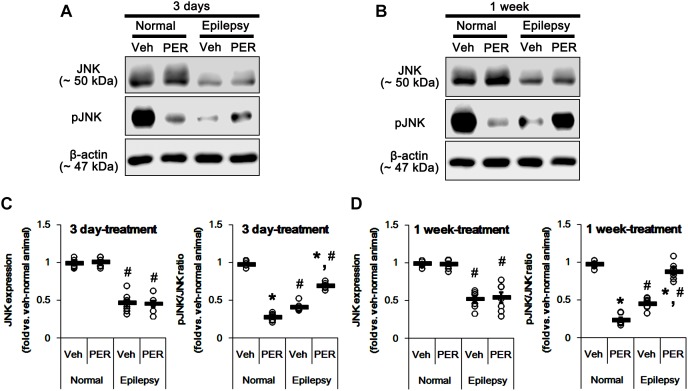
Effects of perampanel on JNK expression and its phosphorylation. **(A,B)** Representative images for western blot of JNK and phospho (p)-JNK in the hippocampal tissues obtained from 3-days **(A)** and 1-week treated groups **(B)**. **(C,D)** Quantifications of JNK and pJNK levels in 3-days **(C)** and 1-week treated groups **(D)**. Open circles indicate each individual value. Horizontal bars indicate mean value. Error bars indicate SEM (*^∗,^*^#^*p* < 0.05 vs. vehicle-treated animals and normal animals, respectively; *n* = 7, respectively).

c-Jun N-terminal kinase is implicated in AMPA receptor tracking during synaptic plasticity in the hippocampus (Zhu et al., 2002). Furthermore, CAMKII and PKA indirectly increase pGluA1-S831 and -S845 phosphorylation via enhancement of JNK phosphorylation, respectively (Ahn and Choe, 2009). Thus, it is likely that perampanel-mediated changes in pGluA1 ratio may be also affected by JNK activity. In normal rats, perampanel reduced pJNK ratios to 28 and 26% of vehicle level in 3-days [*F*_(1,12)_ = 965.18; *p* < 0.05 vs. vehicle, *n* = 7, respectively; Figure 8A,C and Supplementary Figure 6] and 1-week treated group [*F*_(1,12)_ = 540.64; *p* < 0.05 vs. vehicle, *n* = 7, respectively; Figure 8A,C], respectively, without changing its expression level [*F*_(1,12)_ = 0.26 and 0.15, respectively; *n* = 7, respectively; Figure 8A,C].

In epileptic rats, JNK expression and pJNK ratio were decreased to 49 and 44% of vehicle-treated normal rat level [*F*_(1,26)_ = 214.16 and 705.58, respectively; *p* < 0.05, *n* = 14, respectively; Figure 8A,C]. Unlike normal animals, perampanel increased pJNK ratios to ∼ 70 and ∼ 89% of vehicle-treated normal rat level in 3-days [*F*_(1,12)_ = 126.24; *p* < 0.05 vs. vehicle, *n* = 7, respectively; Figure 8A,C] and 1-week treated groups [*F*_(1,12)_ = 66.04; *p* < 0.05 vs. vehicle, *n* = 7, respectively; Figure 8A,C], respectively, without affecting its expression [*F*_(1,12)_ = 0.03 and 0.05, respectively; *n* = 7, respectively; Figure 8A,C].

**FIGURE 9:**
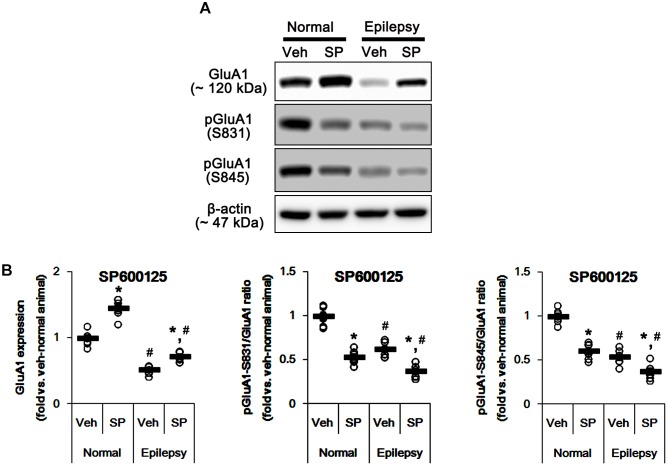
Effects of SP600125 (SP) on GluA expressions and their phosphorylations. **(A)** Representative images for western blot of GluA1, phospho (p)-GluA1-S831, and pGluA1-S845 in the hippocampal tissues obtained from 3-days infused groups. **(B)** Quantifications of GluA1, pGluA1-S831, and pGluA1-S845 levels in SP600125-infused groups. Open circles indicate each individual value. Horizontal bars indicate mean value. Error bars indicate SEM (*^∗,^*^#^*p* < 0.05 vs. vehicle-treated animals and normal animals, respectively; *n* = 7, respectively).

Considering the altered GluA1 phosphorylations in epileptic animals, these findings indicate that JNK activity may be involved in increases in pGluA1-S831 and -S845 ratios. Indeed, SP600125 (a JNK inhibitor) increased GluA1 expression levels in normal [*F*_(1,12)_ = 49.98; *p* < 0.05 vs. vehicle, *n* = 7, respectively; Figure 9A,B and Supplementary Figure 7] and epileptic rats [*F*_(1,12)_ = 29.97; *p* < 0.05 vs. vehicle, *n* = 7, respectively; Figure 9A,B], but reduced pGluA-S831 [*F*_(1,12)_ = 95.25 and 39.77, respectively; *p* < 0.05 vs. vehicle, *n* = 7, respectively; Figure 9A,B] and -S845 ratios [*F*_(1,12)_ = 77.96 and 13.11, respectively; *p* < 0.05 vs. vehicle, *n* = 7, respectively; Figure 9A,B] in both groups. Therefore, our findings indicate that perampanel-induced alteration in JNK activity may regulate GluA1 phosphorylations.

### Effects of Perampanel on Activities of Protein Phosphatases

**FIGURE 10:**
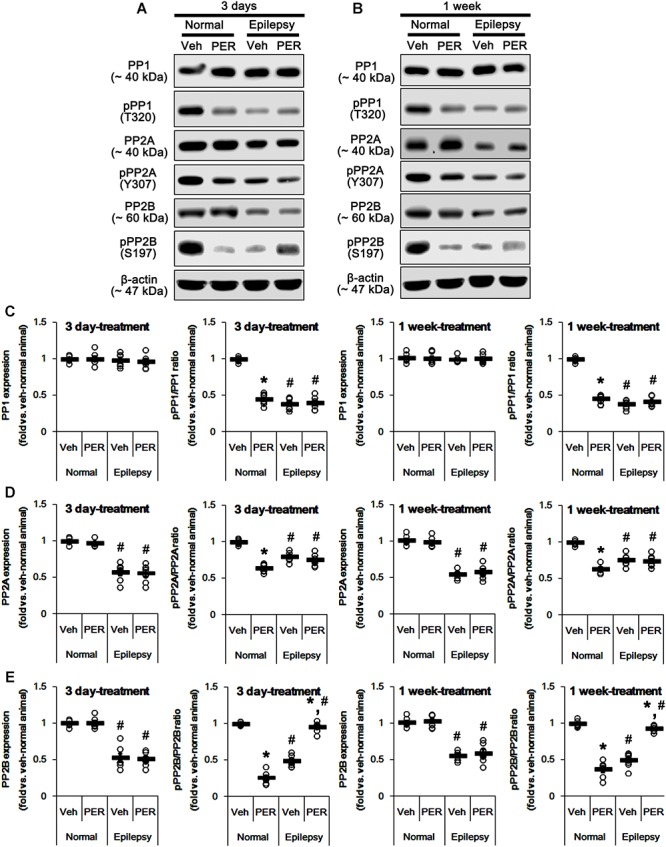
Effects of perampanel on PP1, PP2A, and PP2B expressions and their phosphorylations. **(A,B)** Representative images for western blot of PP1, phospho (p)-PP1, PP2A, pPP2A, PP2B, and pPP2B in the hippocampal tissues obtained from 3-days **(A)** and 1-week treated groups **(B)**. **(C–E)** Quantifications of PP1, pPP1 **(C)**, PP2A, pPP2A **(D)**, PP2B, and pPP2B **(E)** levels in 3-days and 1-week treated groups. Open circles indicate each individual value. Horizontal bars indicate mean value. Error bars indicate SEM (*^∗,^*^#^*p* < 0.05 vs. vehicle-treated animals and normal animals, respectively; *n* = 7, respectively).

GluA1 phosphorylation is also regulated by various PP activities (Kim and Ziff, 2014; Stockwell et al., 2016; Sanderson et al., 2018). In addition, PKA-mediated phosphorylation affects the PP activities (Yan and Surmeier, 1997; Flores-Hernandez et al., 2000; Ahn et al., 2007; Min et al., 2017). Therefore, we tested whether perampanel also affects PP phosphorylations. In normal animals, perampanel reduced pPP1, pPP2A, and pPP2B ratios in 3-days [*F*_(1,12)_ = 343.64, 216.69, and 549.6, respectively; *p* < 0.05 vs. vehicle, *n* = 7, respectively; Figure 10A–E and Supplementary Figure 8] and 1-week treated group [*F*_(1,12)_ = 410.25, 249.56, and 200.63, respectively; *p* < 0.05 vs. vehicle, *n* = 7, respectively; Figure 10A–E], although it did not affect their expression levels (Figure 8B,D 10A–D and Supplementary Figure 6).

In epileptic rats, pPP1 ratio was ∼ 38% of vehicle-treated normal rat level [*F*_(1,26)_ = 1091.25; *p* < 0.05, *n* = 14, respectively; Figure 10B,C], while its expression level was similar to that in normal animals [*F*_(1,26)_ = 0.71; *n* = 14, respectively; Figure 10B,C]. PP2A expression and pPP2A ratio were ∼ 56 and ∼ 77% of vehicle-treated normal rat level [*F*_(1,26)_ = 265.39 and 110.66, respectively; *p* < 0.05, *n* = 14, respectively; Figure 10B,D]. PP2B expression and pPP2B ratio were also reduced to ∼ 55 and ∼ 43% of vehicle-treated normal rat level [*F*_(1,26)_ = 212.14 and 348.26, respectively; *p* < 0.05, *n* = 14, respectively; Figure 10B,E]. Perampanel did not affect PP1 expressions and pPP1 ratios in 3-days [*F*_(1,12)_ = 0.27 and 0.16, respectively; *n* = 7, respectively; Figure 10B,C] and 1-week treated group [*F*_(1,12)_ = 0.1 and 1.13, respectively; *n* = 7, respectively; Figure 10B,C]. Similarly, PP2A expressions and pPP2A ratios were unaltered by 3-days [*F*_(1,12)_ = 0.03 and 1.12, respectively; *n* = 7, respectively; Figure 10B,D] and 1-week perampanel treatment [*F*_(1,12)_ = 0.57 and 0.09, respectively; *n* = 7, respectively; Figure 10B,D]. However, perampanel elevated pPP2B ratios to ∼ 96 and ∼ 93% of vehicle-treated normal rat level in 3-days [*F*_(1,12)_ = 149.13; *p* < 0.05 vs. vehicle, *n* = 7, respectively; Figure 10B,E] and 1-week treated group [*F*_(1,12)_ = 119.25; *p* < 0.05 vs. vehicle, *n* = 7, respectively; Figure 10B,E] without changing its expression [*F*_(1,12)_ = 0.12 and 0.46, respectively; *n* = 7, respectively; Figure 10B,E]. Since the phosphorylations of protein phosphates inhibit their activities (Hashimoto et al., 1988; MacDonnell et al., 2009) and the effects of perampanel on pGluA1-S831 and -S845 ratios, our findings indicate that perampanel may activate PP1, PP2A, and PP2B in normal animals, but inhibit PP2B activity in epileptic rats. Therefore, it is likely that PP2B would be involved in alterations in pGluA1-S831 and -S845 ratios induced by perampanel treatment.

**FIGURE 11:**
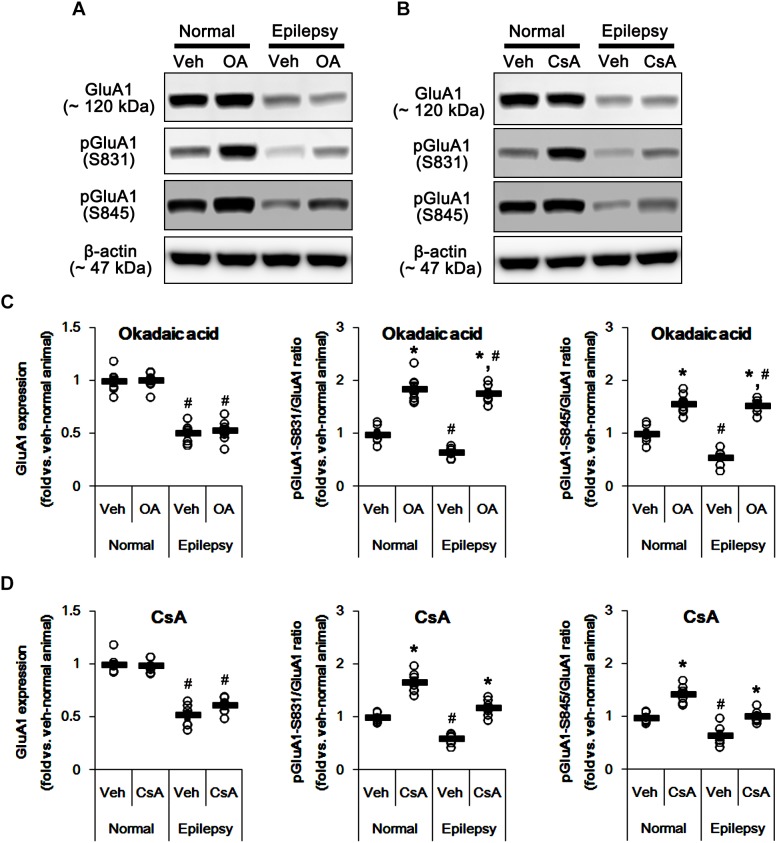
Effects of okadaic acid (OA) and CsA on GluA expressions and their phosphorylations. **(A,B)** Representative images for western blot of GluA1, phospho (p)-GluA1-S831, and pGluA1-S845 in the hippocampal tissues obtained from 3-days infused groups **(B)**. **(C,D)** Quantifications of GluA1, pGluA1-S831, and pGluA1-S845 levels in okadaic acid- **(C)** and CsA-infused groups **(D)**. Open circles indicate each individual value. Horizontal bars indicate mean value. Error bars indicate SEM (*^∗,^*^#^*p* < 0.05 vs. vehicle-treated animals and normal animals, respectively; *n* = 7, respectively).

To confirm this, we applied okadaic acid and (PP1/PP2A inhibitor) and CsA (a PP2B inhibitor) to normal and epileptic rats. Okadaic acid increased pGluA1-S831 and -S845 ratios in normal [*F*_(1,12)_ = 52.44 and 36.14, respectively; *p* < 0.05 vs. vehicle, *n* = 7, respectively; Figure 11A–D and Supplementary Figure 9] and epileptic rats [*F*_(1,12)_ = 235.11 and 177.5, respectively; *p* < 0.05 vs. vehicle, *n* = 7, respectively; Figure 11A–D] without altering GluA1 expression levels [*F*_(1,12)_ = 0.02 and 0.2, respectively; *n* = 7, respectively; Figure 11A–D]. Similarly, CsA increased pGluA1-S831 and -S845 ratios in normal [*F*_(1,12)_ = 66.82 and 39.35, respectively; *p* < 0.05 vs. vehicle, *n* = 7, respectively; Figure 11A–D] and epileptic rats [*F*_(1,12)_ = 73.63 and 19.77, respectively; *p* < 0.05 vs. vehicle, *n* = 7, respectively; Figure 11A–D], while it did not affect GluA1 expression levels in both groups [*F*_(1,12)_ = 0.1 and 3.67, respectively; *n* = 7, respectively; Figure 11A–D]. Although the roles of PP1 and PP2A in GluA1 phosphorylation could not be excluded, these findings suggest that perampanel-induced phosphorylation of PP2B may influence GluA1 phosphorylations in epileptic rats.

### Effect of Perampanel on Spatial Memory in Normal and Epileptic Rats

**FIGURE 12:**
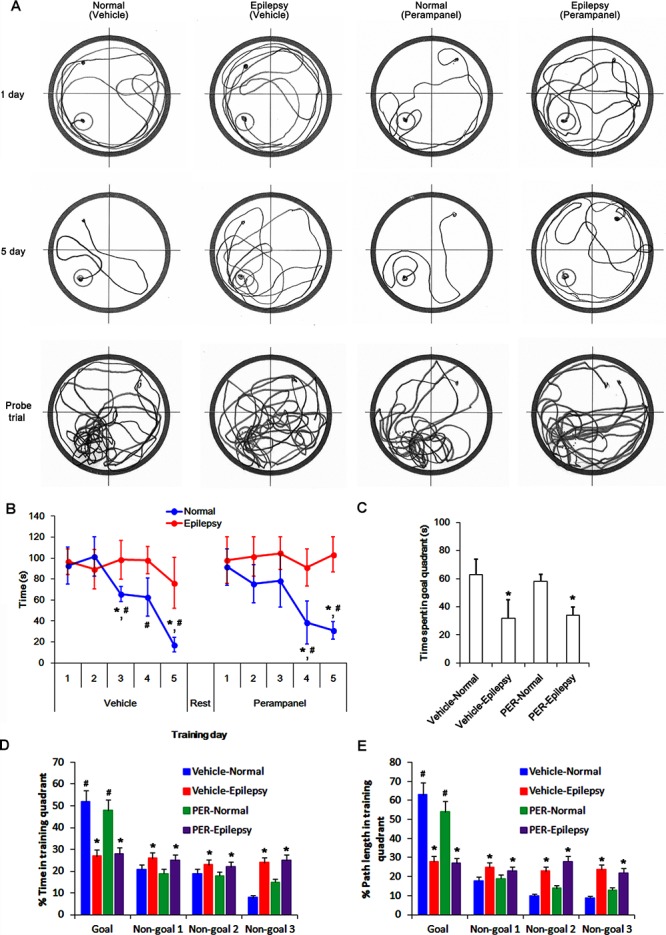
Profiles of behavioral test by Morris water maze. **(A)** Representative traces of swimming plot in Morris water maze test. **(B)** The quantitative analyses of the Morris water maze. Over 5 days of training, normal rats improve their ability to find the submerged platform, which exhibits decreasing escape time, while epileptic rats do not. Perampanel (PER) does not affect escape duration in normal and epileptic rats. ^∗,#^*p* < 0.05 vs. first day and epileptic rats, respectively; *n* = 5, respectively. Error bars in graphs indicates SEM. **(C–E)** The quantitative analyses of the probe trials. Perampanel (PER) does not affect the time spent in goal quadrant **(C)** and the percentage of the time spent **(D)** and the path length **(E)** in goal quadrant in normal and epileptic rats. ^∗,#^*p* < 0.05 vs. normal animals and non-goal quadrants, respectively; *n* = 5, respectively. Error bars in graphs indicates SEM.

To evaluate the cognitive effects of perampanel, we employed the Morris water maze test (Figure 1). Over 5 days of training, normal rats improved their ability to find the submerged platform, which exhibited decreasing escape time [*F*_(1,48)_ = 6.652; *p* < 0.05 vs. epileptic rats, *n* = 5, respectively; Figure 12A,B]. In probe trials, time spent in goal quadrant in normal rats was higher than that in epileptic rats [*F*_(1,8)_ = 16.853; *p* < 0.05 vs. epileptic rats, *n* = 5, respectively; Figure 12A,C]. The percentage of the time spent and the path length in goal quadrant were also higher than those in epileptic rats [*F*_(1,8)_ = 21.14 and 14.136, respectively; *p* < 0.05 vs. epileptic rats, *n* = 5, respectively; Figure 12A,D,E]. Perampanel did not affect escape duration between both groups [*F*_(1,__48)_ = 0.781; *n* = 5, respectively; Figure 12A,B] and time spent in goal quadrant in probe trials in normal rats [*F*_(1,8)_ = 1.635, *n* = 5, respectively; Figure 12A,C] and epileptic rats [*F*_(1,8)_ = 0.965, *n* = 5, respectively; Figure 12A,C]. The percentage of the time spent and the path length in goal quadrant were also unaffected by perampanel in normal rats [*F*_(1,8)_ = 1.4362 and 1.1362, respectively, *n* = 5, respectively; Figure 12A,D,E] and epileptic rats [*F*_(1,8)_ = 1.0659 and 0.8436, respectively, *n* = 5, respectively; Figure 12A,D,E]. No difference in average swimming velocity was observed among all experimental groups. These findings indicate that perampanel may not influence acquisition, consolidation and retention of memory in normal and epileptic rats.

## Discussion

**FIGURE 13:**
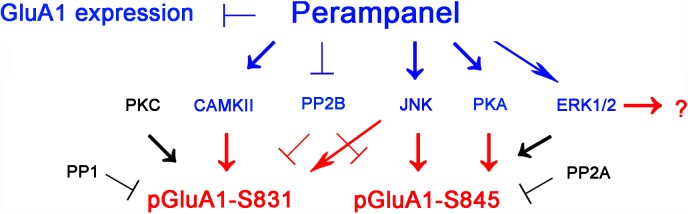
Summary of the effects of perampanel on GluA1 phosphorylations in epileptic rats. Perampanel reduces GluA1 expression. In addition, perampanel increases CAMKII, JNK, PKA, PP2B, and ERK1/2 phosphorylations (blue), which regulate GluA1 phosphorylation (red) except ERK1/2. Perampanel does not affect PKC, PP1, and PP2A phosphorylations (black), which are reported to modulate GluA1 phosphorylations.

The major findings in the present study are that perampanel reduced GluA1 expression and regulated GluA1 phosphorylations by multiple signaling molecules in epileptic rats. Briefly, perampanel increased pCAMKII and pPKA ratios, which phosphorylate GluA1-S831 and -S845 site, respectively. Perampanel also enhanced pJNK and pPP2B ratios, which phosphorylates and dephosphorylates both GluA1-S831 and -S845 sits (Figure 13). Furthermore, these perampanel-induced changes did not lead to a detrimental impact on cognitive ability in normal and epileptic rats.

In the present study, perampanel effectively reduced seizure activities in epileptic rats, although some animals (30% in treated animals) were non-responders. Furthermore, perampanel reduced GluA1 expression in epileptic rats that showed the lower GluA1 expression than control animals. These findings indicate that the anti-epileptic effect of perampanel may be relevant to inhibition of GluA1 expression as well as blockaded of AMPA receptor-mediated currents as previously reported (Hanada et al., 2011; Ceolin et al., 2012; Krauss, 2013; Steinhoff et al., 2013; Chen et al., 2014). The effects of AMPA receptor antagonist on GluA1 expression have been controversial. NBQX, a competitive AMPA receptor antagonist, increases GluA1 expression (Thiagarajan et al., 2005) or not (Maeng et al., 2008). Although we could not explain the underlying mechanisms concerning perampanel-mediated down-regulation of GluA1 expression, it is plausible that the properties of perampanel as a non-competitive (allosteric) AMPA receptor antagonist would result in the distinct effect on GluA1 expression as compared to NBQX. Furthermore, it is considerable that perampanel affected CAMKII and JNK phosphorylations (activities). CAMKII inhibition and gene deletion reduce GluA1 expression level (Ota et al., 2010; Hagihara et al., 2011). In contrast, JNK activation diminishes GluA1 expression level (Lin and Lee, 2012). Indeed, the present data reveal that KN-93 (a CAMKII inhibitor) reduced GluA1 expression, while SP600125 (a JNK inhibitor) increased it in normal and epileptic rats. Therefore, it is likely that perampanel may reduce GluA1 expression in normal and epileptic rats through CAMKII and JNK signaling pathways. Further studies are needed to elucidate the pharmacological mechanisms of perampanel in the regulation of GluA1 expression.

Since AMPA receptor is involved in long-term potentiation (Kessels and Malinow, 2009), blockade of AMPA receptor by perampanel could theoretically result in negative effects on cognition. However, recent clinical studies demonstrate that perampanel does not negatively affect cognition in epilepsy patients (Villanueva et al., 2016; Meschede et al., 2018; Piña-Garza et al., 2018). Similar to human studies, the present study demonstrates that perampanel did not influence spatial learning in normal and epileptic rats during Morris water maze test. Furthermore, perampanel enhanced pGluA-S831 and -S845 ratios, although it reduced GluA1 expression. Regulation of the phosphorylation of serine residues in GluA1 by kinases (PKA, PKC, CaMKII, ERK1/2, and JNK) and phosphatases (PP1, PP2A, and PP2B) plays an important role in governing the conductance and trafficking of AMPA receptor in and out of the synaptic membranes (Lee et al., 1998, 2000; Shepherd and Huganir, 2007). In the present study, perampanel increased pCAMKII and pPKA ratios, which phosphorylate GluA1-S831 and -S845 site, respectively (Banke et al., 2000; Wang et al., 2006; Ahn and Choe, 2009). In addition, perampanel increased pJNK and pPP2B ratios (indicating JNK activation and PP2B inhibition, respectively), which phosphorylates and dephosphorylates both GluA1-S831 and -S845 sits, respectively (Ahn and Choe, 2009; Kim et al., 2009; Kam et al., 2010). Indeed, KN-93, H-89, and SP600125 diminished pGluA1-S831 and -S845 ratios, while CsA increased them. However, BIM and U0126 did not affect GluA1 phosphorylation ratios. With respect to the negative feedback-regulation of AMPA receptor activity in response to AMPA (Snyder et al., 2003), our findings suggest that perampanel may enhance GluA1 phosphorylations via regulating CAMKII, PKA, JNK and PP2B activities in epileptic rats, which may be one of adaptive responses for the diminished GluA1 expression or AMPA receptor-mediated currents. Thus, our findings also provide the possible underlying mechanisms answering why perampanel does not have a detrimental impact on cognitive ability, in spite of blockade of AMPA receptor.

In the present study, pGluA1-S831 ratio in epileptic rats was lower than that in normal animals. Furthermore, pPP1 ratio was lower than that in normal animals, while its expression level was similar to that in normal animals. These findings indicate that PP1 activity in the epileptic hippocampus may be higher than that in the normal one. Since PP1, but not PP2A and PP2B, dephosphorylates GluA1-S831 site (Huang et al., 2001), it is likely that PP1 activation would reduce GluA1-S831 phosphorylation ratio in epileptic animals. Indeed, okadaic acid (a PP1/PP2A inhibitor) increased pGluA1-S831 ratio in the present study. However, perampanel did not affect PP1 expression and pPP1 ratio in epileptic animals. Thus, our findings suggest that perampanel may regulate GluA1-S831 phosphorylation independent of PP1 activity in epileptic hippocampus.

Unlike PP1, the present study shows that PP2A and PP2B expressions and their phosphorylation ratios in epileptic animals were lower than those in normal animals. Similar to the case of pGluA1 ratios, it is likely that the reduced pPP2A and pPP2B ratios may be a compensatory response for maintenance of their activities against down-regulation of expressions. However, perampanel increased pGluA1 ratio in epileptic animals, concomitant with the elevated pPP2B, but not pPP2A, ratio, although okadaic acid and CsA increased pGluA1 ratios in epileptic animals. Therefore, our findings indicate that PP2B may be the potential pharmacological target of perampanel.

In the present study, pERK1/2 ratio in epileptic animals was lower than that in normal animals. Because both PP2A and PP2B deactivates ERK1/2 kinase activity through dephosphorylation of the threonine and tyrosine residues (Waskiewicz and Cooper, 1995; Gabryel et al., 2006; Min et al., 2017), it is likely that PP2A and PP2B activations would affect pGluA1 ratios in epileptic rats via reducing ERK1/2 activity. However, U0126 did not affect pGluA1 ratios, although perampanel elevated them epileptic animals. Therefore, these findings indicate that perampanel-mediated ERK1/2 phosphorylation may not be relevant to GluA1 phosphorylation.

In the present study, there was no difference in the effects of inhibitors of kinases and PPs on GluA1 expressions and its phosphorylation ratios between normal and epileptic rats. Unexpectedly, the present data demonstrate that the responses to perampanel in normal animals were different from those in epileptic rats. Similar to the case of epileptic animals, perampanel reduced GluA1 expression in 3-days and 1-week treated normal animals. In 3-days treated groups, pGluA1-S831 and -S845 ratios were decreased, concomitant with reductions in pPKC, pCAMKII, pPKA, pJNK, pPP1, pPP2A, and pPP2B ratios. In 1-week treated group, however, pGluA1-S831 ratio was rebounded more than vehicle-treated animals, although pPKC, pCAMKII, and pJNK ratios were reduced. Furthermore, pGluA1-S845 ratio was increased to vehicle level, accompanied by recovery of pPKA ratio. Perampanel also reduced pPP1, pPP2A, and pPP2B ratios in 1-week treated groups. Although we could not explain the exact mechanisms in the present study, it can be speculated that these phenomena may be adaptive responses to prolonged inhibition of AMPA receptor functionality in normal hippocampus. Conversely, the down-regulated GluA1 expression or the distinct neurochemical characteristics of epileptic hippocampus would lead to these discrepancies of perampanel actions between normal and epileptic animals. Further studies are needed to elucidate the pharmacological actions of perampanel in normal animals.

In conclusion, to the best of our knowledge, the present data demonstrate previously unreported pharmacological properties of perampanel concerning GluA1 phosphorylation and its up-stream regulatory signaling pathways in normal and epileptic rats. Therefore, our findings suggest that perampanel may regulate AMPA receptor functionality via not only blockade of AMPA receptor but also modulation of GluA1 phosphorylations.

## Author Contributions

T-CK designed and supervised the project. All authors performed the experiments described in the manuscript and analyzed the data. J-EK and T-CK wrote the manuscript.

## Conflict of Interest Statement

The authors declare that the research was conducted in the absence of any commercial or financial relationships that could be construed as a potential conflict of interest.
