# Supplementary material for: Perampanel Affects Up-Stream Regulatory Signaling Pathways of GluA1 Phosphorylation in Normal and Epileptic Rats
Source: Front Cell Neurosci. 2019 Mar 1;13:80. doi: 10.3389/fncel.2019.00080 (PMC6405474; doi:10.3389/fncel.2019.00080)
Supplement: Supplementary file 1 [file Data_Sheet_1.PDF]

Supporting information

Perampanel affects up-stream regulatory signaling pathways of GluA1 phosphorylation in normal and epileptic rats

Ji-Eun Kim[1,3], Hui-Chul Choi[2,3], Hong-Ki Song[2,3], Tae-Cheon, Kang[1,3*]

[1]Department of Anatomy and Neurobiology, College of Medicine, Hallym University, Chunchon 24252, South Korea

[2]Department of Neurology, College of Medicine, Hallym University, Chunchon 24252, South Korea

[3]Institute of Epilepsy Research, College of Medicine, Hallym University, Chunchon 24252, South Korea

* Correspondence should be addressed to T-C K (e-mail: tckang@hallym.ac.kr)

Supplementary Table 1. Primary antibodies used in the present study

Antigen	Host	Manufacturer (catalog number)	Dilution used
CaMKII	Rabbit	Santa Cruz (sc-13082)	1:1,000
ERK	Rabbit	Biorbyt (Orb160960)	1:2,000
GluA1	Mouse	Synaptic system (182011)	1:500
JNK	Rabbit	Protein tech (10023-1-AP)	1:1,000
pCaMKII	Rabbit	Abcam (ab32678)	1:5,000
pERK	Rabbit	Bioss (bs-3330R)	1:1,000
pGluA1-S831	Rabbit	Abcam (ab109464)	1:5,000
pGluA1-S845	Rabbit	Millipore (AB5849)	1:1,000
pJNK	Rabbit	Millipore (#07-105)	1:1,000
PKA-catalytic	Rabbit	BioVision (3115-100)	1:1,000
PKC	Rabbit	Abcam (ab23511)	1:1,000
PP1	Rabbit	Abcam (ab52619)	1:5,000
PP2A	Rabbit	Cell Signaling (#2038)	1:5,000
PP2B	Rabbit	Millipore (07-068-I)	1:1,000
pPKA-catalytic	Rabbit	Assay Biotec (A0548)	1:1,000
pPKC	Rabbit	Abcam (ab59411)	1:1,000
pPP1	Rabbit	Abcam (ab62334)	1:5,000
pPP2A	Rabbit	Sigma (SAB4503975)	1:1,000
pPP2B	Rabbit	Badrilla (A010-80)	1:1,000
β-actin	Mouse	Sigma (A5316)	1:5,000

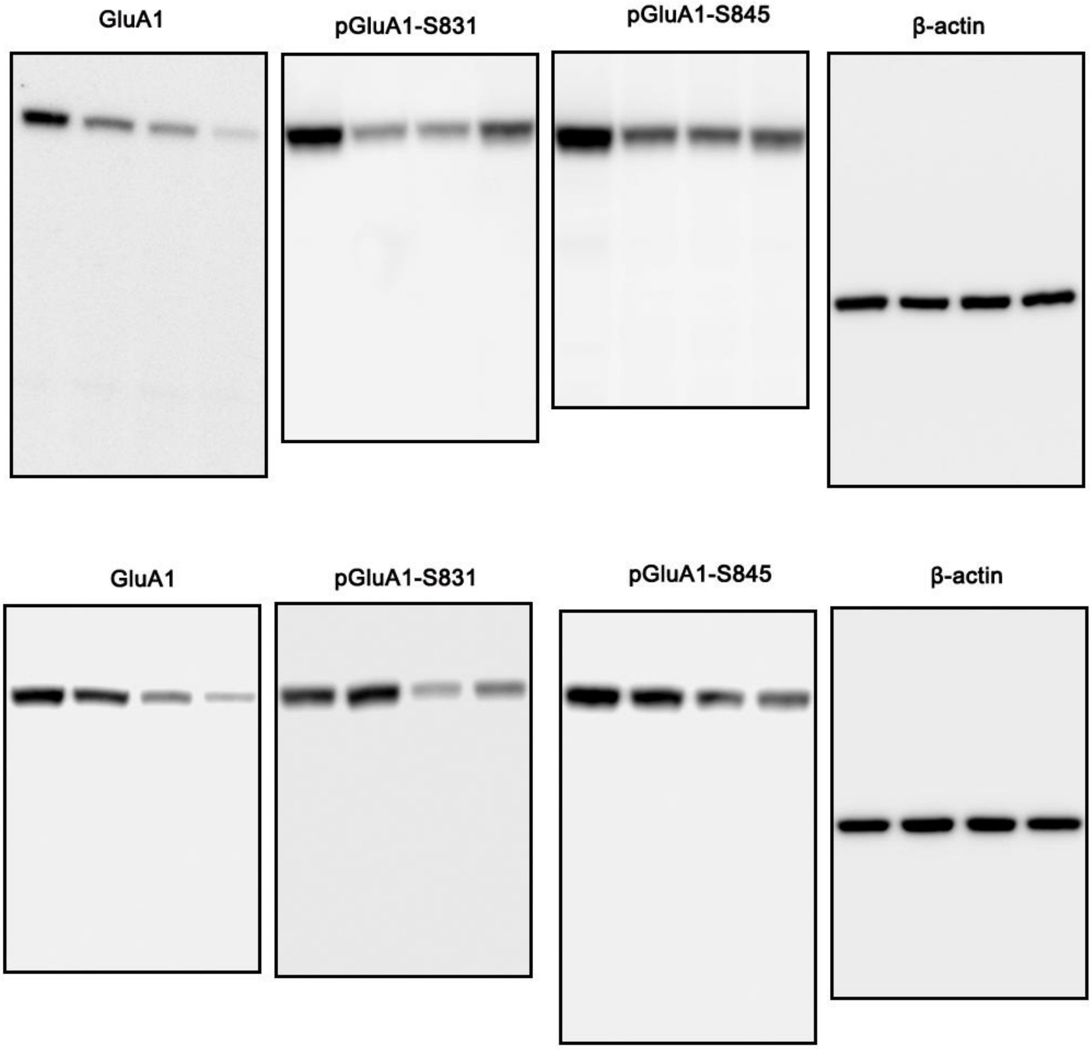

Supplementary Fig. 1. Full-length gel images of western blot data in Fig. 3.

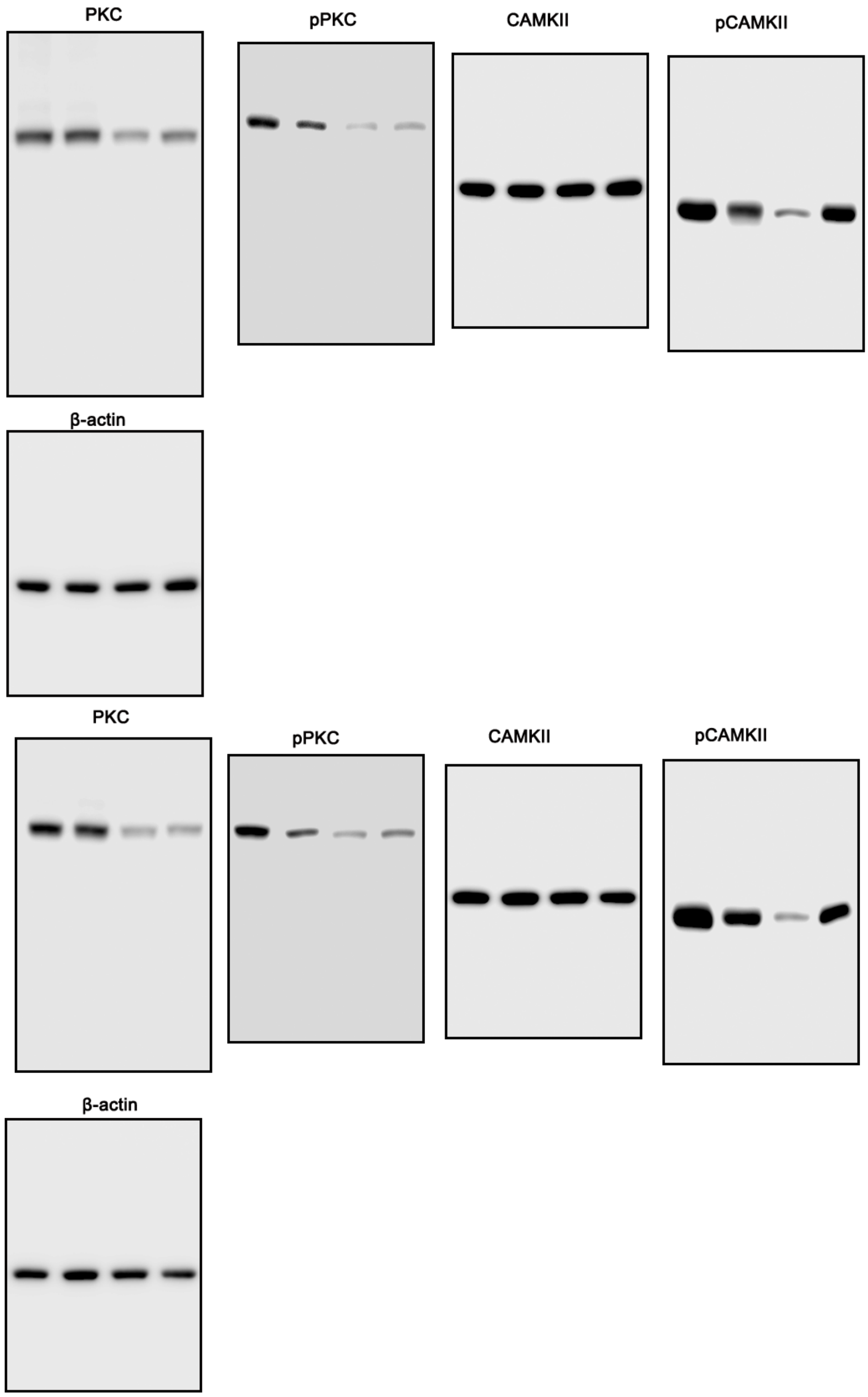

Supplementary Fig. 2. Full-length gel images of western blot data in Fig. 4.

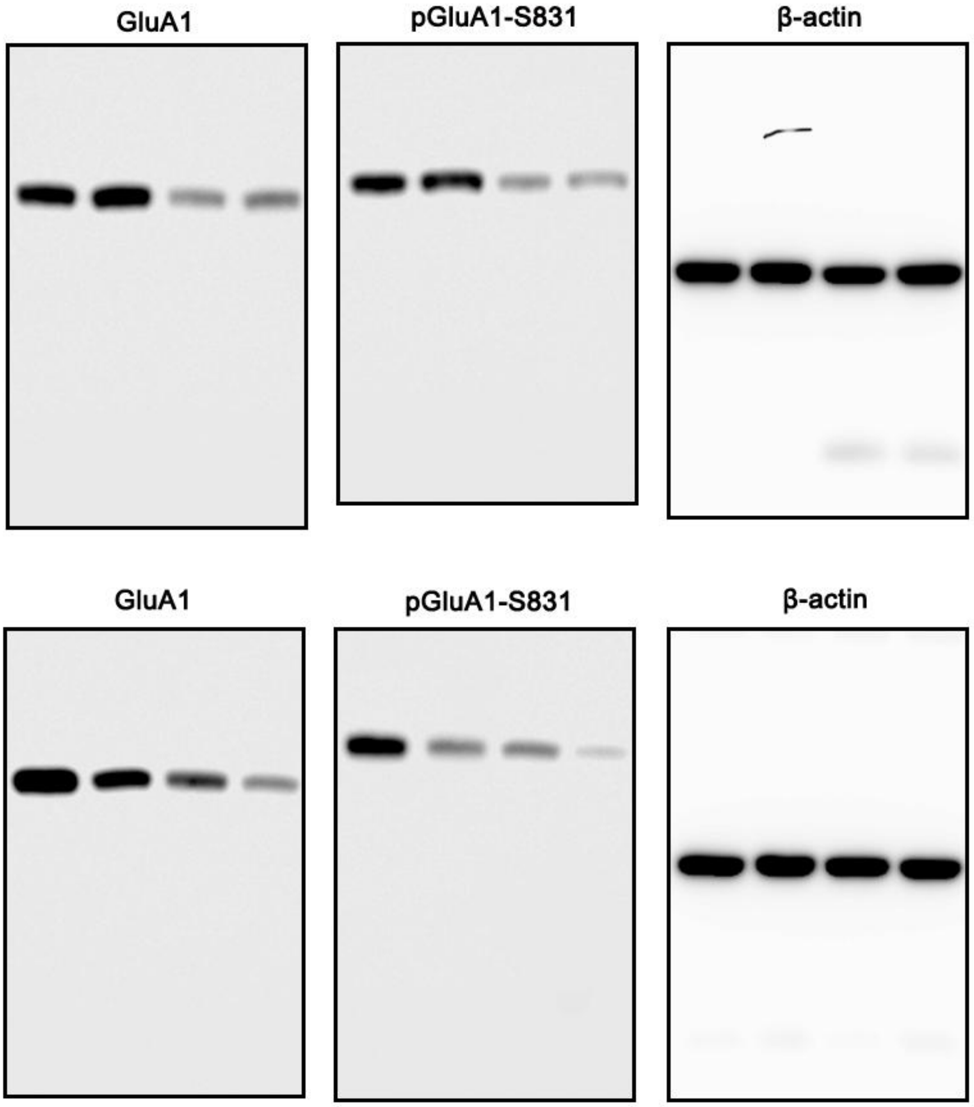

Supplementary Fig. 3. Full-length gel images of western blot data in Fig. 5.

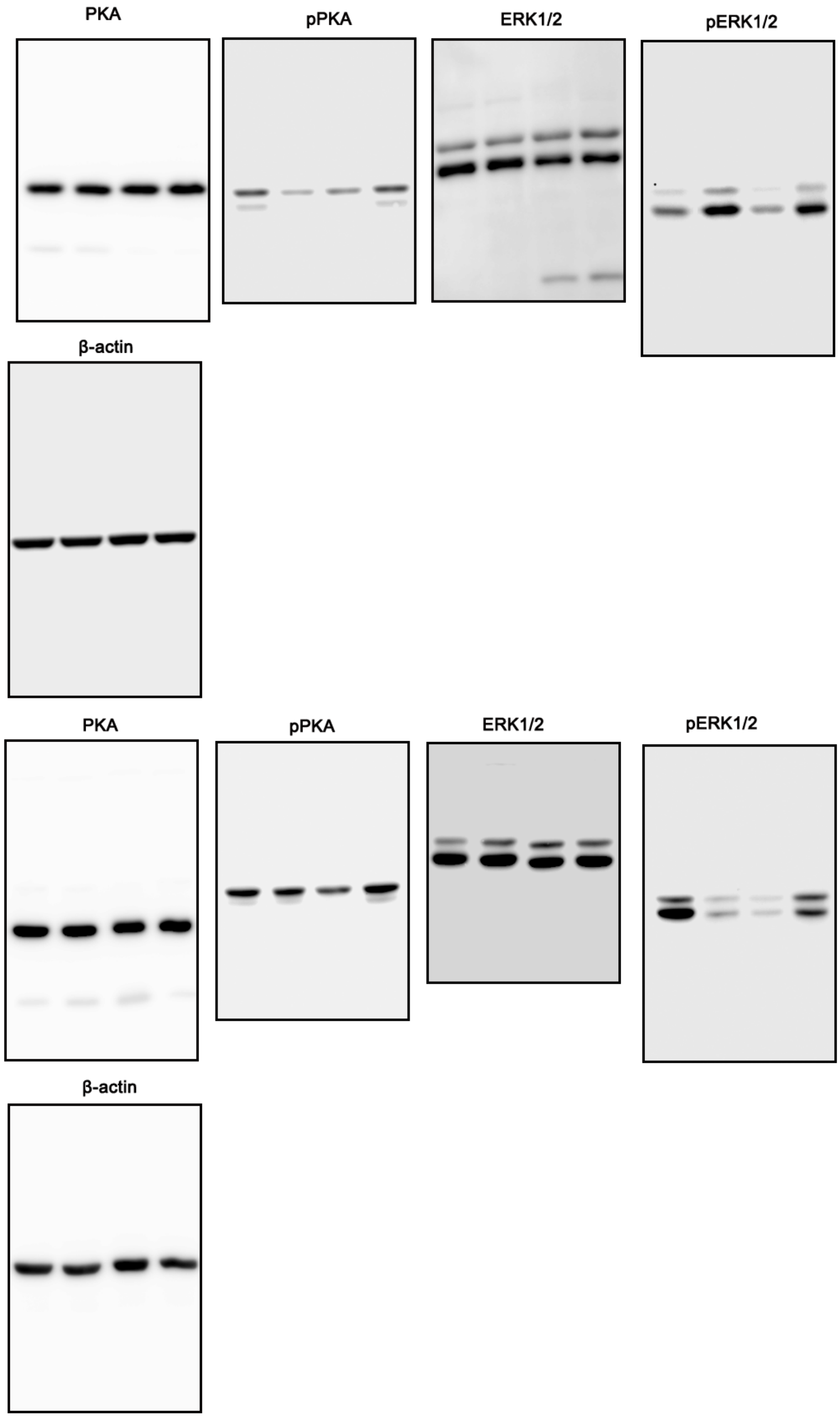

Supplementary Fig. 4. Full-length gel images of western blot data in Fig. 6.

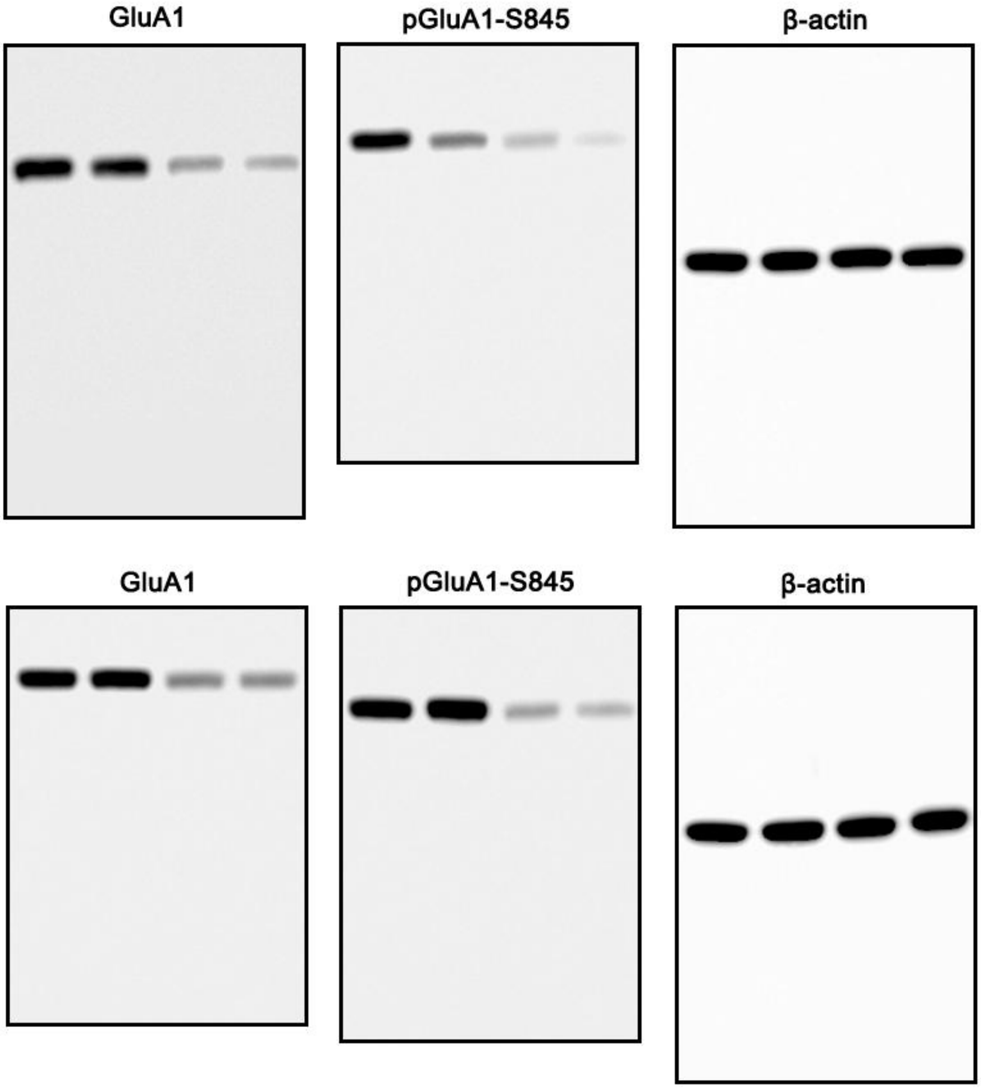

Supplementary Fig. 5. Full-length gel images of western blot data in Fig. 7.

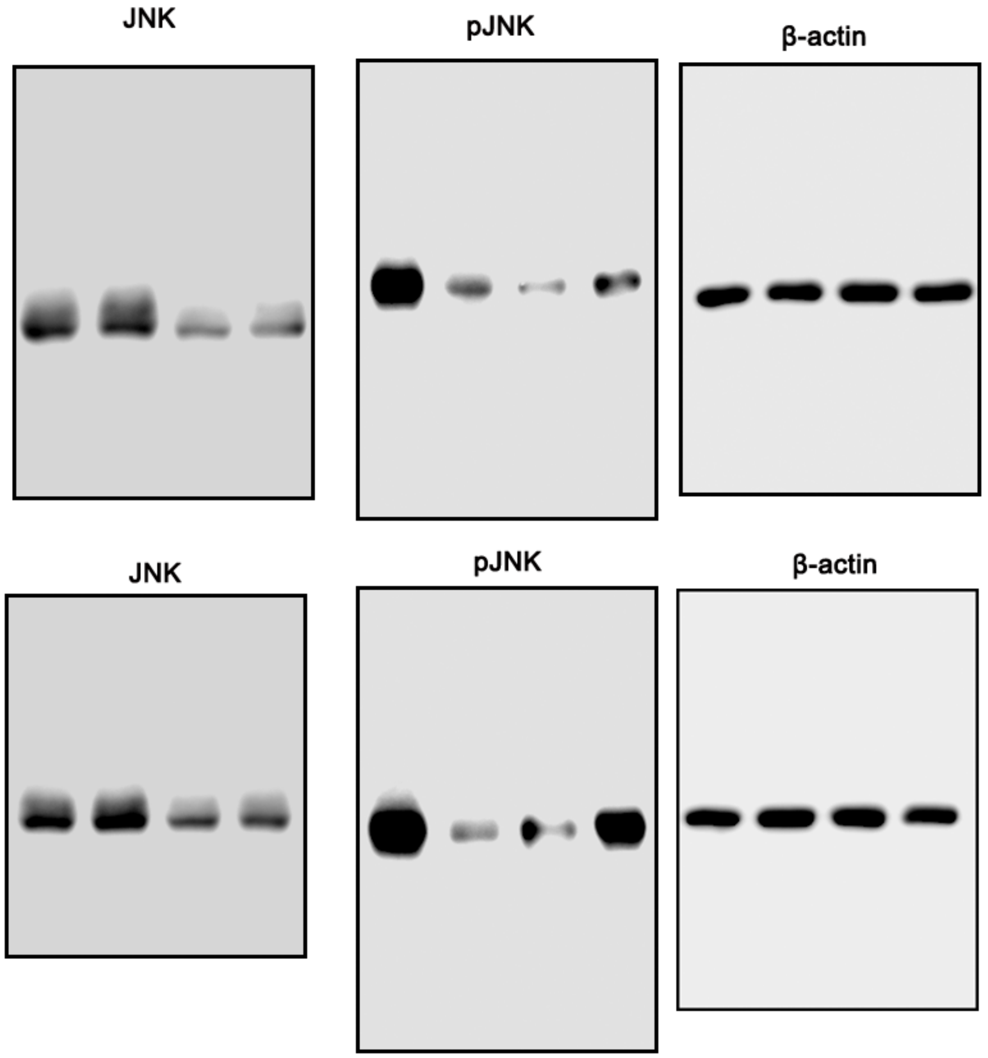

Supplementary Fig. 6. Full-length gel images of western blot data in Fig. 8.

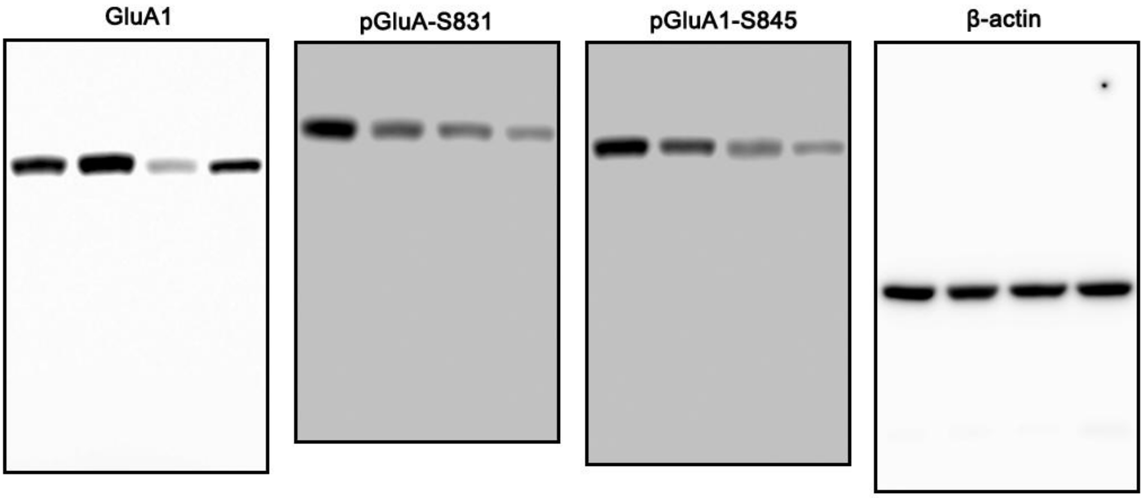

Supplementary Fig. 7. Full-length gel images of western blot data in Fig. 9.

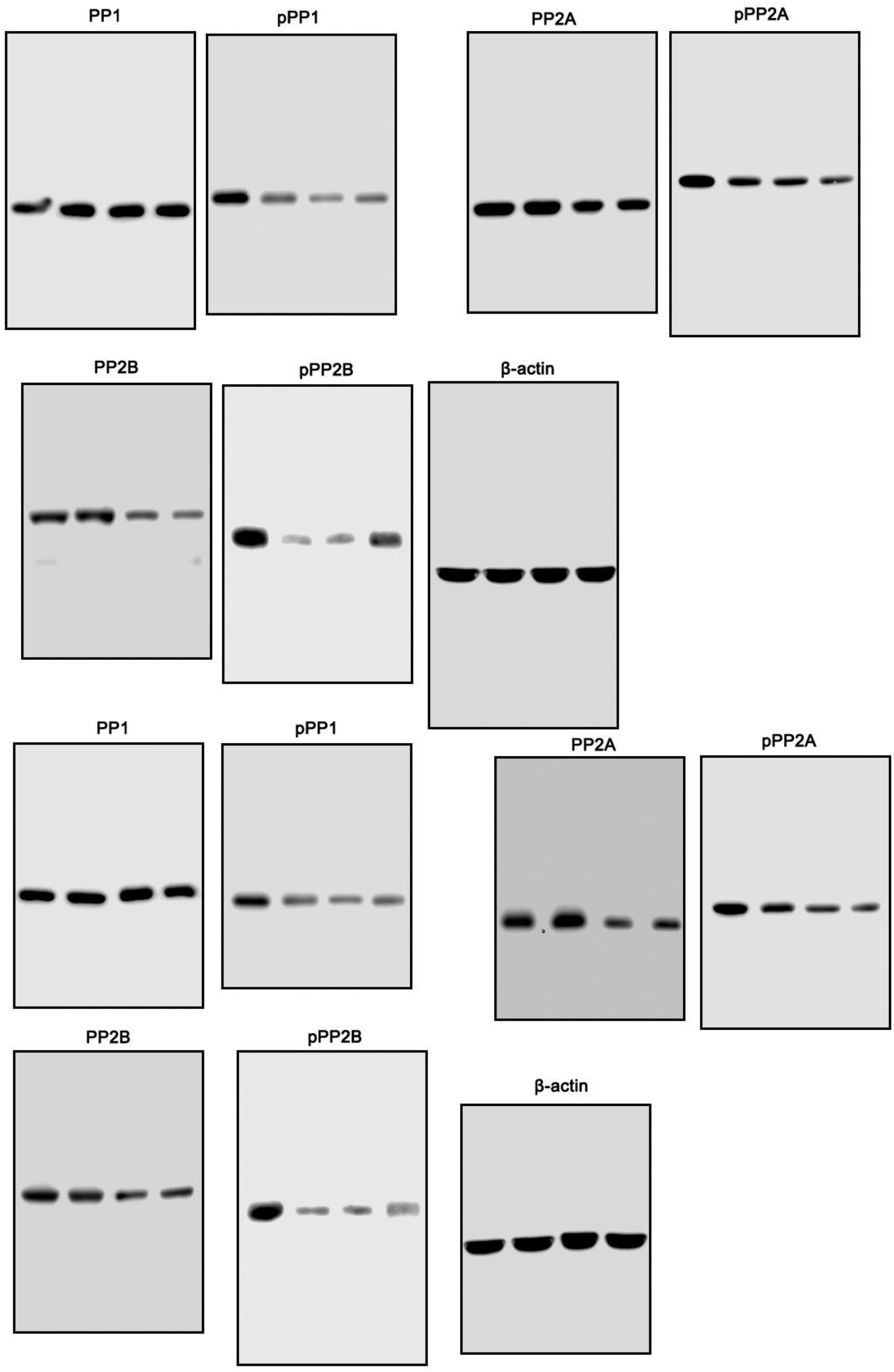

Supplementary Fig. 8. Full-length gel images of western blot data in Fig. 10.

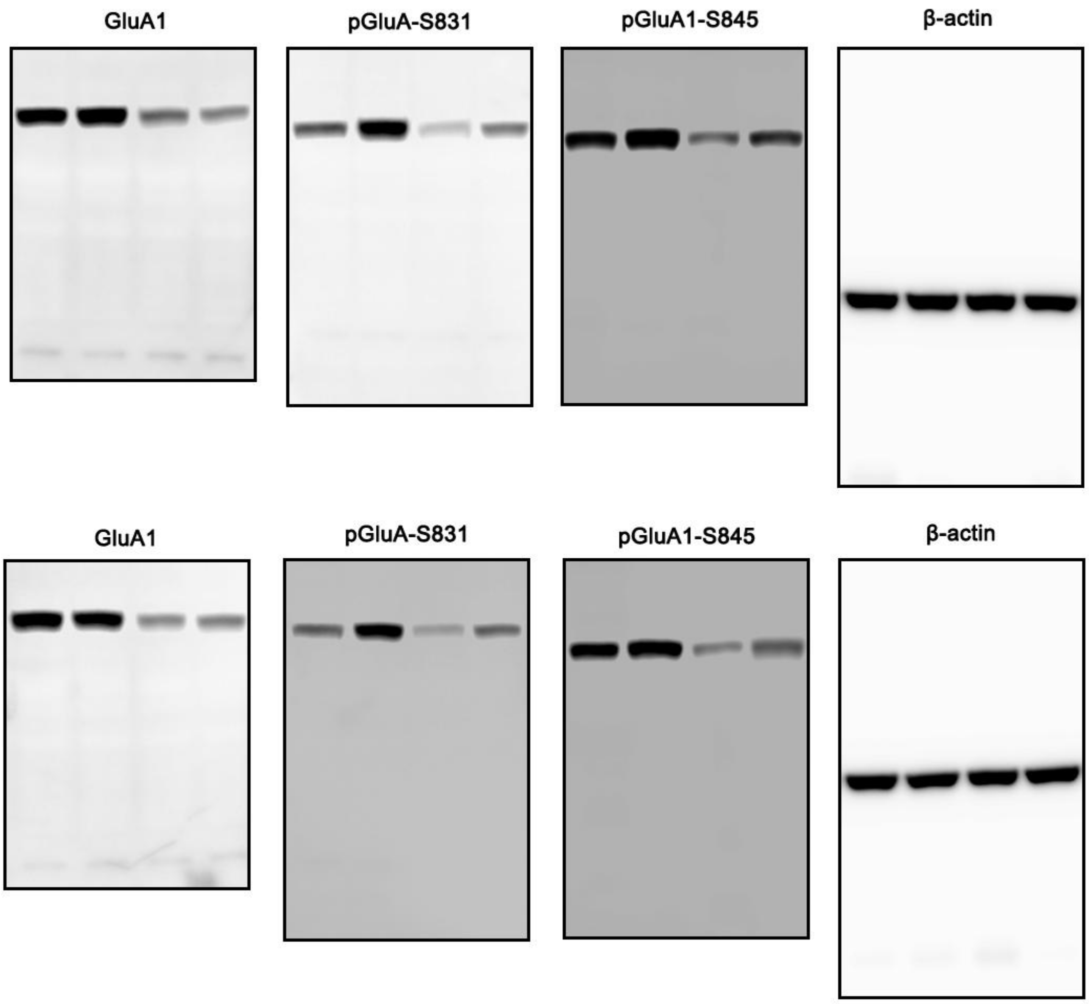

Supplementary Fig. 9. Full-length gel images of western blot data in Fig. 11.
